# Activated Carbon Prepared from Waste Coffee Grounds: Characterization and Adsorption Properties of Dyes

**DOI:** 10.3390/ma17133078

**Published:** 2024-06-22

**Authors:** Feryelle Aouay, Afef Attia, Lasâad Dammak, Raja Ben Amar, Andre Deratani

**Affiliations:** 1 Research Unit “Advanced Technologies for Environment and Smart Cities”, Faculty of Sciences, University of Sfax, 3000 Sfax, Tunisiaafef.attia@fss.usf.tn (A.A.); 2 Institut Europeen des Membranes, IEM UMR-5635, CNRS, ENSCM, University Montpellier, Place Eugène Bataillon, 34095 Montpellier, France; 3 Institut de Chimie et des Matériaux Paris Est, ICMPE UMR-CNRS 7182-UPEC, Université Paris Est Creteil 2 rue Henri Dunant, 94320 Thiais, France; dammak@u-pec.fr

**Keywords:** dye removal, AO7, activated carbon, spent coffee grounds, adsorption process, experimental design methodology, optimization

## Abstract

Spent coffee grounds (SCGs) have great potential as a useful, value-added biological material. In this context, activated carbon (AC) was prepared from SCGs by an activation process using H_3_PO_4_ at 600 °C in the air and used as an adsorbent for the azo dye AO7, a model molecule for dye colorants found in textile industry effluents. X-ray diffraction, SEM and BET revealed that the AC was predominantly amorphous, consisting of a powder of 20–100 µm particles with mesopores averaging 5.5 nm in pore size. Adsorption kinetics followed a pseudo-second-order law, while the Langmuir model best fitted the experimental isotherm data (maximum capacity of 119.5 mg AO7 per AC g). The thermodynamic parameters revealed that adsorption was endothermic and spontaneous. All the characterizations indicated that adsorption occurred by physisorption via mainly π–π interactions. The best experimental removal efficiency optimized by means of a Box–Behnken design and response surface methodology was 98% for an initial AO7 concentration of 20 mg·L^−1^ at pH 7.5 with a dose of 0.285 g·L^−1^ of AC and a contact time of 40 min. These results clearly show that activated carbon prepared from SCGs can be a useful material for efficiently removing organic matter from aqueous solutions.

## 1. Introduction

Water is an essential resource which integrity is often compromised by the discharge of industrial waste containing harmful substances into natural environments [1]. A striking example of this challenge is the treatment of contaminated industrial wastewater, particularly from the textile industry, one of the biggest water consumers [2]. This industry generates a large volume of waste loaded with dyes and chemicals, posing a threat to human health and the environment [3]. It is responsible for the largest amount of wastewater compared to other industries, at over 56% [4,5]. Recent estimates have indicated an annual consumption of more than 10,000 tons of over 100,000 commercial dyes [6,7,8,9]. Approximately 10–15% of the dye is lost during the dyeing process, and around 20% of the lost dye ends up in wastewater [10,11], with a concentration of up to 1 mg·L^−1^ [12,13]. Synthetic dyes, especially azo dyes, are of particular concern because of their carcinogenic, mutagenic and teratogenic properties, as well as the high chemical and biological oxygen demand required for their degradation [14,15]. Acid Orange 7 (AO7), an azo dye widely used as a model molecule, exemplifies these challenges. Despite its water solubility and stability, AO7 can cause methemoglobinemia upon human exposure, resulting in symptoms such as cyanosis and weakness [15,16,17].

Azo dyes, characterized by an azo chromophore group (-N=N-) and an electron-receiving sulfonate group (-SO_3_^−^), are difficult to degrade by conventional processing methods [18,19,20]. In fact, textile dyes are not easily biodegradable, requiring the development of specific microbial strains and immobilized enzymes [21]. Another approach to decontaminating water polluted by textile dyes is the use of physicochemical techniques, including electrochemical oxidation [22], coagulation-flocculation [23], filtration [24,25] and photodegradation [26]. However, these methods often have drawbacks, such as high costs and the production of toxic by-products. Adsorption appears to be a promising alternative approach due to its cost-effectiveness and its ability to remove contaminants without introducing additional toxicity. A number of different approaches have been developed [27,28,29]. Activated carbon (AC) is emerging as an adsorbent of choice, especially when derived from natural biopolymers or waste products, offering a sustainable and cost-effective solution for water purification [8]. The production of AC from inexpensive feedstocks available in large quantities makes it possible to envisage an easily scalable adsorption process [30]. In this work, spent coffee grounds (SCGs) were chosen as a precursor to produce AC [31,32].

Coffee is one of the world’s most popular beverages. Every year, more than five million tons of coffee are produced worldwide (International Coffee Organization, London, UK, 2022). Generally, SCGs are disposed of by composting, incineration or landfill. It has been reported that these methods can have a negative impact on the environment, releasing contaminating residues of tannin, caffeine and polyphenols [33]. Other ways of valorizing SCGs have been proposed, such as converting them into biofuel, biodiesel, bioethanol or biomaterials [34] and producing biochar and AC [35,36,37]. These routes can help reinforce the circular economy approach by enabling continuous processes that reduce the accumulation of waste by transforming it into useful, value-added materials while promoting more environmentally friendly models [38,39].

This study examined the removal of AO7, as a model molecule for azo dye used in textile industry, by adsorption onto AC produced from SCGs. The physicochemical properties of the AC produced were investigated using techniques such as TGA-DTG, XRD, FT-IR and SEM. With a view toward optimizing the process, studies including adsorption kinetics and isotherms, as well as thermodynamic characteristics, were carried out to understand how certain experimental parameters such as the dye concentration/adsorbent quantity ratio and contact time can affect the adsorption operation. A statistical analysis was then applied to assess the weight of each of the experimental parameters. The response surface methodology (RSM) model was used to analyze the interactions among the parameters studied in order to gain a better insight into their combined effects. This method has the advantage of evaluating several parameters at once, which reduces the number of experiments required and saves time compared to the traditional method of examining one factor at a time [40,41]. In addition, the efficiency of experimental systems can be effectively evaluated and process variability minimized.

## 2. Materials and Methods

### 2.1. Chemicals

The SCGs used in the present study were collected from local coffee shops and served as a low-cost precursor for AC. Phosphoric acid (H_3_PO_4_, 50%) was used as a chemical activating agent in the preparation of AC. The azo dye AO7 (analytical grade ≥ 85%), which characteristics are given in Table 1, was supplied by Sigma-Aldrich (St. Louis, MO, USA). All aqueous solutions were prepared with deionized water and pH-adjusted with sodium hydroxide (NaOH, 0.2 M) and hydrochloric acid (HCl, 0.2 M).

### 2.2. Activated Carbon Preparation

Two batches of SCG-based AC were prepared according to the following procedure, adapted from [42]. Typically, SCGs (30 g) were washed with deionized water, then dried in an oven at 60 °C. The resulting dried SCGs were immersed in a 50% solution of H_3_PO_4_ in a weight ratio of 1:3 (SCG:H_3_PO_4_) and stirred at 80 °C for 3 h. Slow pyrolysis was then carried out at 600 °C for 2 h in a muffle furnace in the air. The resulting AC was washed with deionized water to a neutral pH and then finally dried in an electric oven at 105 °C for 24 h.

### 2.3. Characterization of AC

The produced AC was characterized using FTIR (Spectrum 100, Perkin Elmer, Waltham, MA, USA) and SEM (Quanta-250, Thermo Fisher Scientific, Waltham, MA, USA) at an accelerating voltage of 20 kV to identify the surface functional groups and morphologies, respectively. The specific surface area, total pore volume and pore diameter were measured using the Brunauer, Emmett and Teller (BET) method (ASAP 2020, Micromeritics, Norcross, GA, USA). The crystalline structure was determined by X-ray diffraction (XRD 6000, Shimadzu, Kyoto, Japan) using Cu Kα1 (λ = 1.5046 Å) with an X-ray source through the 2θ range from 4° to 80°. Thermogravimetric analysis (TGA; Labsys evo1150, Setaram, Caluire-et-Cuire, France) was carried out to determine the thermal stability.

The zero charge point is the pH at which the AC surface becomes neutral. This value (pH_PZC_) was determined using the following procedure: 50 mL of NaCl solution (0.01 M) with pH values ranging from 1 to 10 was prepared by adding NaOH solution (0.2 M) and HCl solution (0.2 M), then 100 mg of AC was added to each solution and left to stir for 48 h at room temperature. The final pH after 48 h was recorded to calculate the pH change. The pH_PZC_ point was identified at the intersection of the curve (final pH—initial pH) and the straight line (final pH = initial pH) using a graphical representation of the data.

### 2.4. Batch Adsorption Studies

A stock solution was prepared at 0.1 g·L^−1^ from AO7 as purchased. The concentrations required for the adsorption experiments were obtained by dilution with deionized water. The desired amount of AC, depending on the experimental conditions chosen, was added to the 100 mL dye solution under magnetic stirring (500 rpm) at room temperature (about 25 °C). The pH was 7.5 and remained unchanged during the adsorption experiments. Once the adsorption process was complete, the solution mixture was passed through a 0.45 μm syringe filter. The filtrate obtained was then analyzed using a UV–Visible spectrophotometer at a wavelength of 483 nm to determine the percentage of dye removal according to the calibration curve. The dye adsorption capacity and removal efficiency of the adsorbent were quantified using Equations (1) and (2), respectively.

(1) $$q_{t}=\frac{C_{0}-C_{t}}{M}\cdot V$$

(2) $$R=\frac{C_{0}-C_{t}}{C_{0}}\cdot 100$$

where *q_t_* (mg·g^−1^) is the adsorption capacity at time t, *R* the AO7 dye removal efficiency, *C*_0_ the initial dye concentration, *C_t_* (mg·L^−1^) the dye concentration at time t, *V* (mL) the solution volume and *M* (g), the mass of the AC adsorbent.

### 2.5. Adsorption Kinetics

The kinetics of the adsorption process were evaluated by varying the contact time between AO7 and AC. The experiment was carried out in a 200 mL suspension with a dye concentration of 30 mg·L^−1^ and 0.6 g AC. The concentration of dye in the solution was determined every 10 min until equilibrium was reached, when its value remained constant. The data were then fitted to pseudo-first-order and pseudo-second-order models (equations presented in Table 2).

### 2.6. Adsorption Isotherms

Adsorption isotherm models can be used to highlight the interactions of AO7 molecules with adsorption sites on the AC surface. This experiment examined the correlation between the amount of dye adsorbed at equilibrium and the amount of sorbent used. For this study, the dye concentrations ranged from 10 to 90 mg·L^−1^, while the AC quantity and contact time were kept constant and equal to 0.3 g·L^−1^ and 60 min, respectively. Data adequacy and characterization of the adsorbent surfaces as heterogeneous or homogeneous were assessed using Langmuir and Freundlich isotherm models (linearized equations presented in Table 3).

### 2.7. Adsorption Thermodynamics

Equilibrium adsorption isotherms were performed at three different temperatures (298, 321 and 339 K) to determine the thermodynamic parameters, such as enthalpy, entropy and Gibbs free energy, using Equations (7)–(9) presented in Table 4.

### 2.8. Optimization of AO7 Removal by AC Adsorption

A Box–Benken quadratic three-factor response RSM model was developed to determine the impact of three independent variables on AO7 dye removal: contact time (*X*_1_), initial dye concentration (*X*_2_) and adsorbent dose *(X*_3_). This approach made it possible to optimize the response variable (*Y*) as a function of the input variables while reducing the amount of experimental work required for the adsorption studies. Design-Expert v12 software (Stat-Ease, Minneapolis, MI, USA) was applied to analyze the data using the RSM approach. In this study, the box in which the selected parameters were varied was 10–60 min for the contact time, 10–90 mg·L^−1^ for the initial dye concentration and 0.1–0.4 g·L^−1^ for the adsorbent dose. Fifteen experiments were designed to optimize the combination of input variables, with AO7 dye removal efficiency (%) used as the output response. To assess the model fit, analysis of variance (ANOVA) was used. The results derived from the experimental design, structured in three levels, were fitted using a model designed to capture the desired response. A classical polynomial regression equation was used to express the predicted response (*Y*):

(10) $$Y\left(\%\right)={\;a}_{0}+\sum a_{i}X_{i}+\sum \sum a_{ij}X_{i}X_{j}+\sum a_{ii}X_{i}^{2},\;i\neq j$$

where the *X_i_* is the input factors that can influence the response *Y*, *n* the number of variables, *a*_0_ the constant intercept, *a_i_* the linear and *a_ii_* the quadratic coefficient (with (*i* = 1, 2… n) and *a_ij_* (*i* = 1, 2… n − 1; *j* = 2, … n) the interaction coefficient between two input factors. The regression and RSM models were used to investigate the highest projected dye removal.

## 3. Results and Discussion

Two batches of AC were prepared. Very similar results were obtained in terms of material characterization and adsorption properties.

### 3.1. Characteristics of the Prepared Activated Carbon

#### 3.1.1. Chemical Group Analysis

**Figure 1:**
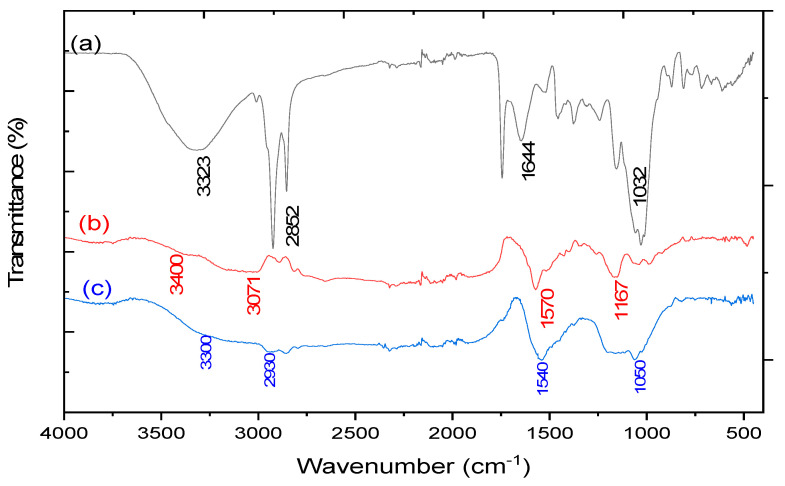
The FTIR spectrum for SCGs (**a**). AC before (**b**) and after dye adsorption (**c**).

The raw SCG mixture and the AC produced were compared using the FTIR technique to highlight the change in characteristic functional groups during activation (Figure 1a,b). The following band assignments were made based on the data found in the literature [43,44,45]:

- The presence in the SCG spectrum (Figure 1a) of a broad transmission band between 3200 and 3600 cm^−1^ was attributed to the O-H stretching vibrations in cellulose, hemicellulose and lignin molecules. This band almost disappeared in the AC spectrum (Figure 1b) due to dehydration caused by the combination of H_3_PO_4_ and heat action.
- The sharp bands at 2800–3000 cm^−1^ can be assigned to stretch vibrations of the C-H bond in the saturated hydrocarbons. These strong absorptions in the SCG spectrum have been assumed due to the presence of components other than cellulose, hemicellulose and lignin molecules [44]. These bands disappeared in the AC spectrum, probably indicating their decomposition during pyrolysis.
- The bands between 1650 and 1750 cm^−1^ characteristic of carbonyl groups in the SCG spectrum revealed the presence of lignin and the strong absorption observed in the 1000–1200 cm^−1^ region linked to C-O stretching vibrations of cellulose, hemicellulose and lignin.
- As expected, AC showed stronger absorption than the starting SCG material around 1600 cm^−1^ corresponding to the C=C vibrations in aromatic rings due to pyrolysis. On the other hand, the broad band around 1200 cm^−1^ was attributed to P=O and P-O-C vibrations associated with H_3_PO_4_ activation.

Comparison of the AC spectra before and after dye adsorption (Figure 1b,c) revealed only slight changes due to a strong overlap of their respective characteristic bands. However, an increase in absorption in the 1500–1550 cm^−1^ range linked to the vibrations of azo groups (-N=N-) was observed, confirming the AO7 adsorption.

#### 3.1.2. Structural Characterization

**Figure 2:**
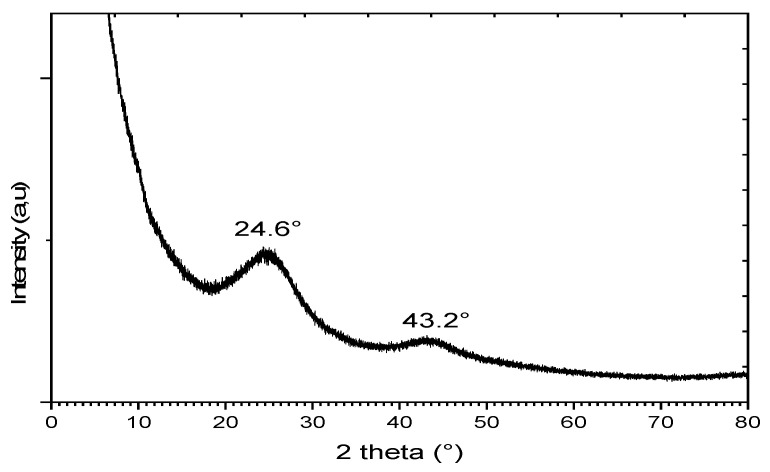
XRD pattern for the AC prepared from SCGs.

The X-ray diffraction was used to study the existence of crystalline structures and the amorphous nature within the AC matrix (Figure 2). The powder spectrum obtained revealed two diffraction peaks at about 2θ = 24 and 44°, as is usually observed for AC prepared from different sources, indicating that the activation process was successful [42,46,47,48]. The peaks observed are generally associated with the (002) and (100) reflection planes of graphite. However, as can be seen, the diffraction peaks are quite broad, suggesting that the structure of the prepared AC consisted of small crystalline phases dispersed in large disordered zones.

The amorphous structure of adsorbent is expected to create a favorable microenvironment for the adsorption of molecules in large quantities with a stronger bond. In contrast, crystalline structures have uniform shapes, making the adsorption ability difficult. The AC prepared from SCGs in this work presented a structure suitable for having good adsorption capability.

#### 3.1.3. Morphological Analysis

**Figure 3:**
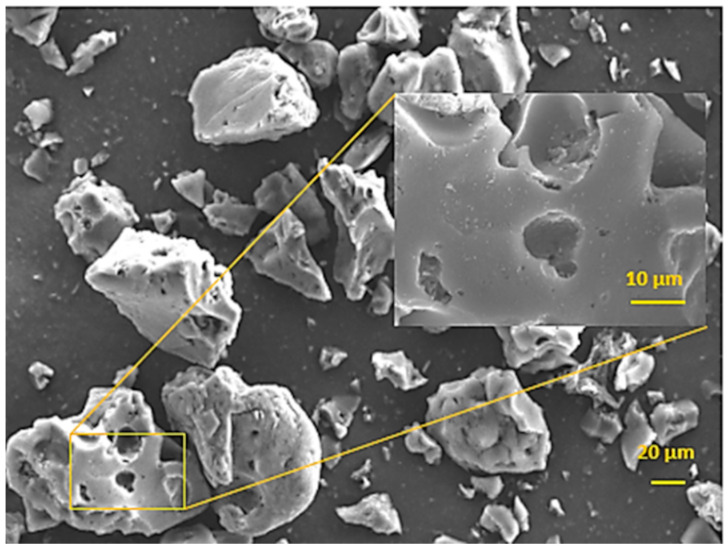
SEM surface image of AC prepared from SCGs.

SEM analysis provided useful information on the shape, size and structure of the AC powder particles (Figure 3). The irregular-shaped particles showed a polydisperse size distribution with dimensions ranging mainly from about 20 to 100 µm. In addition, SEM images enabled us to characterize the porous structure generated during the acid activation and pyrolysis processes. The outer surface of the particles had a macroporous morphology with cavity smaller than 20 µm. The enlargement in Figure 3 shows that interconnection by smaller pores in the cavities ensures the penetration and transfer of low-molecular-weight molecules from the surface to the inner microporosity of the adsorbent.

#### 3.1.4. Textural Properties

**Figure 4:**
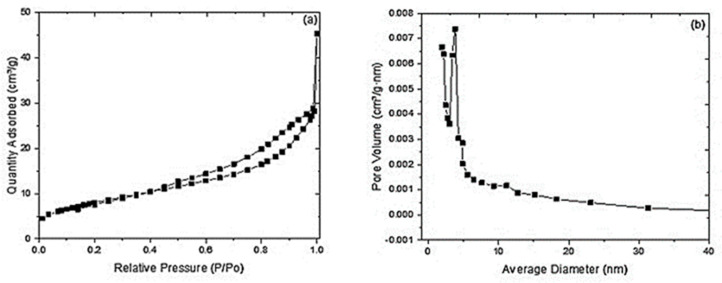
(**a**) N_2_ adsorption/desorption isotherms. (**b**) Pore size distribution of AC.

Activated carbons are porous substances with a wide variety of pores ranging from macropores (>50 nm) to mesopores (2–50 nm) and occasionally a small number of micropores (<2 nm), depending on the activation process [29]. Gas adsorption—in particular, nitrogen adsorption at 77 K—is a commonly used method for studying the characteristics of porous activated carbons. Figure 4a,b show the hysteresis of the adsorption isotherm and the pore distribution deduced for the AC sample, respectively. The material mainly exhibited a mesoporous structure according to IUPAC standards (Figure 4a; Type IV isotherm) [49], with a surface area and pore volume at 295 K of 29.38 m^2^·g and 0.067 cm^3^·g, respectively (Table 5). These values are easily explained by the reduction in the surface area and total volume of AC pores due to pyrolysis, which leads to pore collapse in composite materials [50]. However, given the small molecular size of the dyes, and of AO7 in particular (Table 1), the mean pore size (*D*_m_) of 5.5 nm appears large enough to allow internal mass transfer of these compounds within the AC porous structure.

#### 3.1.5. Thermal Stability

**Figure 5:**
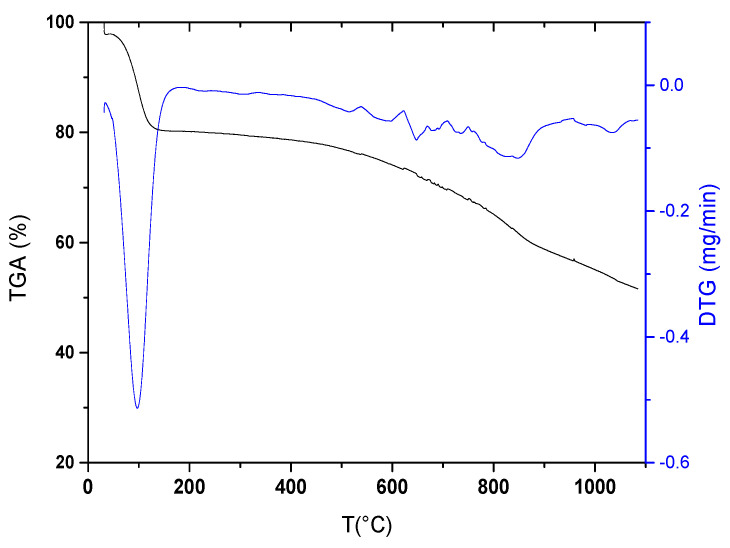
TGA and DTG curves of AC prepared from SCGs.

Figure 5 shows the variation in AC weight between 50 and 1100 °C using TGA. The thermogram obtained displays that a significant mass loss, equivalent to about 20%, occurred from 50 °C to 150 °C. It mainly corresponded to the removal of moisture (DTG peak at 100 °C) and, possibly, of volatile compounds that accumulated within the porous structure of the material during its storage [51]. This result is indicative of the excellent adsorption properties of AC prepared in this work. Then, the curve shows a plateau up to 450 °C with no significant mass loss, revealing high thermal resistance and possible applications in this temperature range. After this second region, a gradual weight loss of about 30% is observed from 450 °C to 1100 °C. This mass decrease is due to decomposition of the oxygenated functions and partial degradation of the carbon skeleton [52,53].

#### 3.1.6. pH Determination of Zero Charge Point

**Figure 6:**
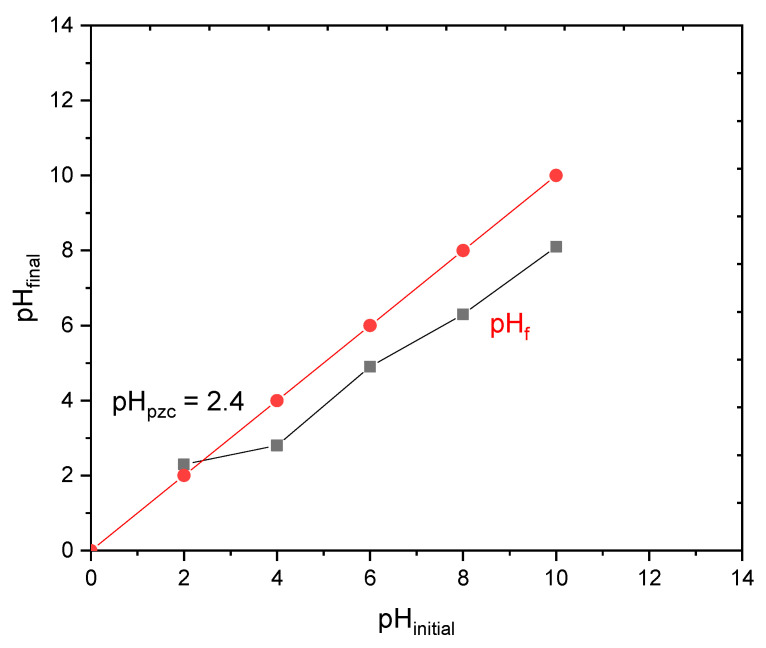
Determination of the point of zero charge pH.

The surface charge of an adsorbent as a function of pH is an important parameter for the adsorption process, as it depends on the chemical groups present on the surface. Figure 6 presents the shift in pH AC suspension compared to the corresponding blank solutions. The intersection of curves at pH 2.4 indicates when the net AC surface charge becomes neutral at this value (pH_PZC_). This implies that, for pH above this, the number of negatively charged surface functions is higher than that of positively charged functions, mainly due to deprotonation of the carboxylic and phenolic groups [53].

### 3.2. Dye Adsorption Characterization

#### 3.2.1. Adsorption Kinetics

**Figure 7:**
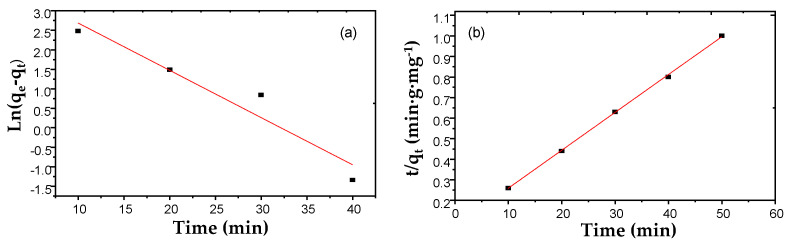
Kinetic plots and fitted kinetic model curve for AO7 adsorption on AC: (**a**) pseudo-first-order kinetic and (**b**) pseudo-second-order models.

Adsorption kinetics and analysis of the quantity adsorbed at equilibrium are the two key parameters for understanding and optimizing the adsorption process. The rate of removal of the AO7 dye from solution by AC was studied in order to establish the kinetic laws of adsorption. These experimental data were modeled in Figure 7 using two theoretical approaches: the pseudo-first-order (PFO) model and the pseudo-second-order (PSO) model (Table 2).

The best fitting for each of the two Equations (3) and (4) were obtained using the coefficients listed in Table 6. It was found that experimental data fitted the PSO model (Figure 7b) much better than the PFO model (Figure 7a). The quantities of dye adsorbed on AC (*q_exp_*) were almost identical to those calculated (R^2^ values close to unity). These results confirm the relevance of the PSO model to describe the adsorption kinetics of the AO7 dye on the AC surface and agree fully with previously reported data for the adsorption of various dyes on activated carbon [29].

#### 3.2.2. Adsorption Isotherms

**Figure 8:**
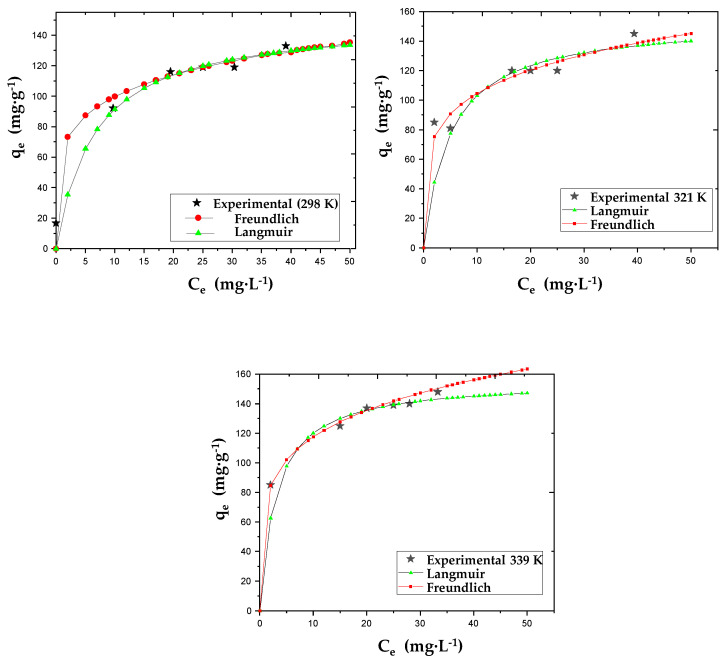
Non-linearized data of the AO7 adsorption isotherm on AC at three different temperatures and the corresponding fitted theoretical curves using the Langmuir and Freundlich models.

Isotherm adsorption modeling makes it possible to determine the various equilibrium parameters, understand the degree of affinity and interpret interaction mechanisms between the adsorbent and adsorbate. Numerous models have been developed in the literature, taking into account parameters such as the number of layers of adsorbed molecules, surface heterogeneity and binding energies [29]. Langmuir and Freundlich isotherm models are the most frequently used due to their simplicity and consistency. The adsorption mechanism of AO7 by AC was analyzed at three different temperatures using these two models. Figure 8 presents the data non-linearized and the corresponding fitted theoretical curves. The optimized parameter values obtained for Equations (5) and (6) are listed in Table 7.

The experimental results fit better with the Langmuir model based on homogeneous monolayer sorption (R^2^ = 0.9877) than with the Freundlich model based on heterogeneous multilayer sorption at all temperatures. As a result, AO7 molecules are assumed to disperse into monolayers on adsorption sites that are identical in terms of energy and without electrostatic interactions between them [18]. The parameter R_L_ is a measure of the separation factor between the adsorbate and adsorbent in a system. A value between 0 and 1 indicates a favorable adsorption process, while R_L_ > 1 represents unfavorable adsorption. When R_L_ = 1, adsorption is linear, and when its value is zero, the adsorption process is irreversible. The separation constant obtained by modeling in Table 7 (R_L_ = 0.178 − 0.223 << 1) suggests a very strong but still reversible adsorption of AO7 by the AC prepared in this work.

#### 3.2.3. Adsorption Thermodynamics

Thermodynamic factors, including standard free energy (*ΔG°*), enthalpy (*ΔH°*) and entropy (*ΔS°*), are the key parameters in understanding the adsorption reaction. It is then possible to gain deeper insight by identifying the spontaneity, heat exchange and degree of disorder of the process. Table 8 presents the values obtained in the temperature range 298–339 K using Equations (7)–(9).

The results led to the following conclusions:

The negative *ΔG°* values indicate that the adsorption process is favorable and spontaneous within the selected temperature range. The *ΔG°* values become more negative with the increasing temperature, suggesting that the adsorption process is facilitated. This effect could be due to the widening of the AC pores with the increasing temperature, resulting in an easier diffusion of AO7 molecules and greater accessibility to adsorption sites located within the AC [52,54].

The positive *ΔS°* value indicates that the AO7 adsorption process is characterized by increased disorder in the interaction between dye molecules and the AC surface. A similar result has been reported for AO7 adsorption on AC from algae [18]. Adsorption is generally considered to decrease the number of degrees of freedom by reducing the adsorbate movement, resulting in a negative *ΔS°* value. The A07 molecule is hydrophobic by nature (Table 1) and is assumed to be solubilized in water as small micellar aggregates. It can therefore be assumed that adsorption of an isolated molecule on the AC interface increases the degree of freedom, which may explain the positive value of *ΔS°* obtained.

The positive *ΔH°* value indicates that the dye adsorption process is endothermic in nature. Based on the *ΔH°* values, it can be deduced that the adsorption is predominantly physisorption through interactions between AO7 molecules and AC active sites such as hydrogen bonds and π–π interactions. This is confirmed by the fact that the *ΔH°* value is less than 40 kJ·mol^−1^ [55,56].

### 3.3. Adsorption Mechanism

**Figure 9:**
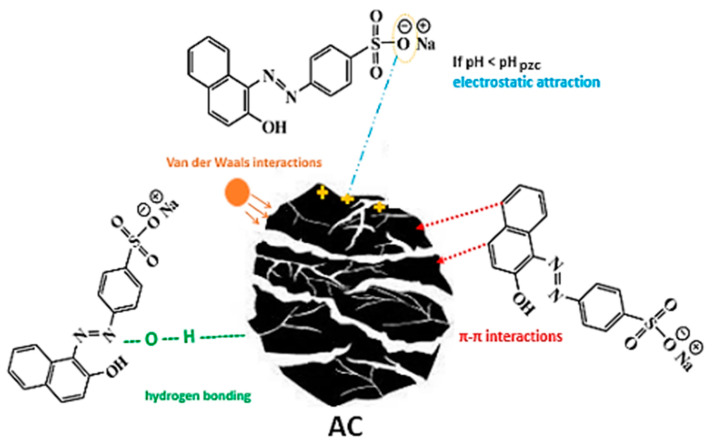
Proposed adsorption mechanisms of AO7 dye onto the activated carbon (AC).

Previous studies have shown that dye adsorption on activated carbon is influenced by various factors, such as electrostatic attraction, hydrogen bonding, Van der Waals interactions and π–π interactions. Interestingly, adsorption of the anionic dye occurs at pH values higher than the pH_PZC_ of AC, while its surface charge is, on average, negative. Under these operating conditions, the predominant electrical interactions are repulsive, making adsorption unfavorable or limited even if a number of positive charges are present. This means that electrostatic interactions cannot be the main mechanism of AO7 adsorption and suggests the involvement of other types of interactions [18]. One possible mechanism is hydrogen bonding, as shown by the analysis of the FTIR spectra (Figure 1). The characteristic peak for hydroxyl groups showed a red shift (move from 3400 cm^−1^ to 3300 cm^−1^) and broadening after AO7 adsorption, pointing to hydrogen bonding between the hydroxyl groups on the AC surface and AO7. In addition, π–π interactions between the adsorbents and dye molecules seem to play a crucial role in the AO7 adsorption process. This is also supported by the FTIR analysis, which revealed a weakening of the absorption intensity and a broadening of the characteristic C=C peak (1600 cm^−1^) in the AC spectrum after AO7 adsorption (Figure 1), suggesting an interaction between the π-electron system of the AC structure and the aromatic rings of the AO7 dye molecules (Table 1) [55,56]. This is in perfect agreement with the endothermic nature of adsorption and the *ΔH°* value marking a physisorption mechanism and π–π-type interactions, as mentioned above. Figure 9 presents the proposed interaction mechanisms occurring during the AO7 adsorption on AC prepared from SCGs in this work.

### 3.4. Experimental Design and Response Surface Plots of AO7 Removal

Preliminary experiments showed that contact time, dye concentration and adsorbent dose were the key factors to be optimized. Although pH and temperature also had an influence, they were not of primary importance for the adsorption of AO7 on the AC prepared in this work. Therefore, to save time, these two parameters were kept at 7.5 and about 25 °C, while only the first three parameters mentioned were varied.

#### 3.4.1. Statistical Analysis of the Derived Response Surface Model

In order to determine the optimum experimental conditions for AO7 removal efficiency by AC (output variable response) and to elucidate the weight of the different input parameters (*X*_1_: contact time, *X*_2_: dye concentration, and *X*_3_: adsorbent dose), batch adsorption experiments were carried out using the Box–Behnken design method as part of the RSM model. A series of 15 experiments was conducted according to a customized design, and the experimental- and model-predicted responses are summarized in Table 9. The last column shows the residual calculated as the difference between the experimental responses and those predicted by the model. Depending on the value of the input factors, the percentage of dye removal varies between 15 and 98%, with residual values well below 4%.

Table 10 presents the analysis of variance (ANOVA) of the regression parameters of the quadratic response surface models predicted for the percent of dye removal. The *F*-value of 66.94 and the adjusted regression coefficient above 99% indicate that the model equation is highly significant. Furthermore, there is only a small probability of 0.01% that this *F*-value is due to noise [52].

This analysis also makes it possible to determine the weight of the input parameters and their interactions through the polynomial coefficients of the model (Table 11). It appears from the *F*-values that *X*_1_, *X*_2_, *X*_3_, *X*_1_*X*_3_, *X*_2_*X*_3_, *X*_1_^2^ and *X*_3_^2^ are the most significant terms in the model. In addition, *p*-values greater than 0.100 indicate that *X*_1_*X*_2_ and *X*_2_^2^ do not significantly contribute to the removal efficiency of AO7. Consequently, the polynomial Equation (10) can simply be written as follows:

(11) $$Y(\%)=78.02+6.54\;X_{1}-26.49\;X_{2}+11.4\;X_{3}+7.75\;X_{1}X_{3}+12.8\;X_{2}X_{3}-10.63\;X_{1}^{2}-7.64\;X_{3}^{2}$$

For the purpose of process optimization, Equation (11) of the model shows the extent to which dye removal efficiency is affected by the various input factors considered through their linear and quadratic terms, as well as their mutual interactions. Negative coefficients indicate that the corresponding factors have a negative impact on the removal efficiency [32]. The factor (*X*_2_) is the most influential parameter: the higher the dye concentration, the lower the removal efficiency (*Y*). Contact time (*X*_1_) and adsorbent dose (*X*_3_) have antagonistic effects.

**Figure 10:**
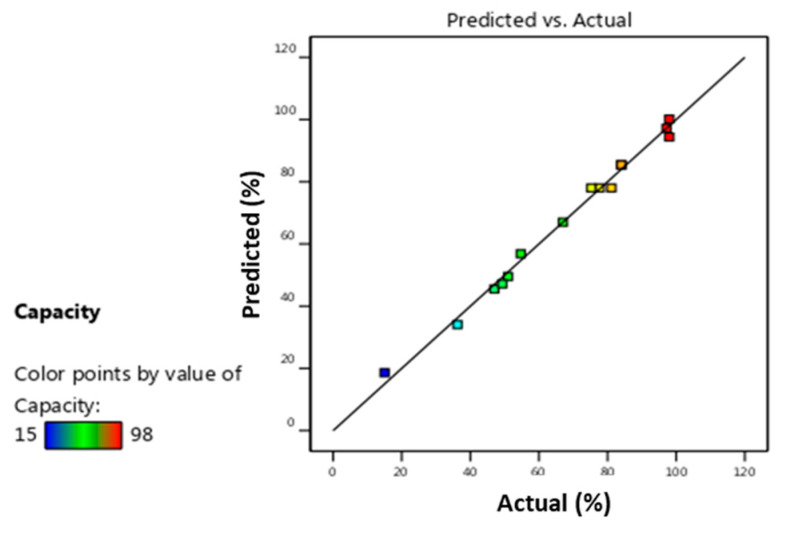
The removal efficiency predicted from Equation (11) (*Y*) versus the actual data (*R*) from Equation (2) for AO7 adsorption using AC.

As mentioned above, the regression coefficient R^2^ obtained in this study was 0.9918, indicating that the developed customized model accounted for about 99% of the variability observed in the experimental data. In addition, the adjusted regression coefficient R^2^_adj_ was 0.9770, which implies that nearly 98% of the variability, given the number of variables and degrees of freedom, can be explained by the model. Figure 10 compares actual values with predicted model-derived responses for AO7 removal, illustrating the fit between the optimization model and experimental data. The proximity of the data points to the diagonal line in the plot of actual versus predicted values and indicates the suitability of the model developed. This means that the statistical model effectively captures the correlation among the three factors investigated in AO7 removal. It was concluded that the Equation (11) model is highly predictive and appropriate to accurately represent the relationship between the variables considered [40].

#### 3.4.2. Interaction Effect of Variables on Dye Removal Efficiency of AO7 Dye by AC

The 3D response surface plots and contour graphs for the removal efficiency of AO7 when using AC give a graphical interpretation of the second-order model equation to examine the relationship between the different factors considered in the adsorption process.

**Figure 11:**
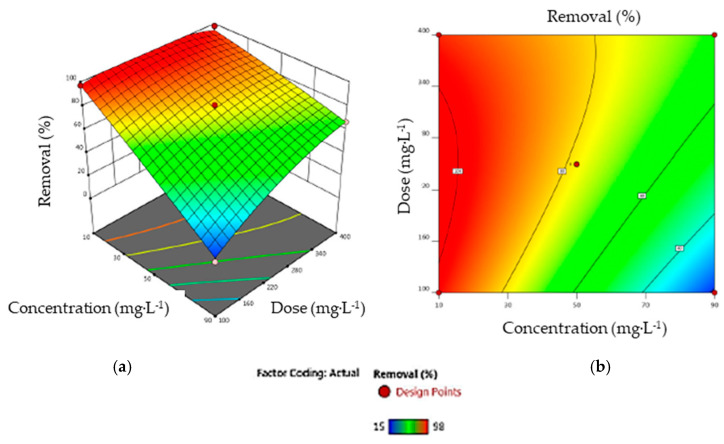
(**a**) The response surface and (**b**) the contour map showing the interaction of AO7 concentration and AC dosage (input parameters *X*_2_ and *X*_3_ in Equation (11), respectively) on dye removal.

**Figure 12:**
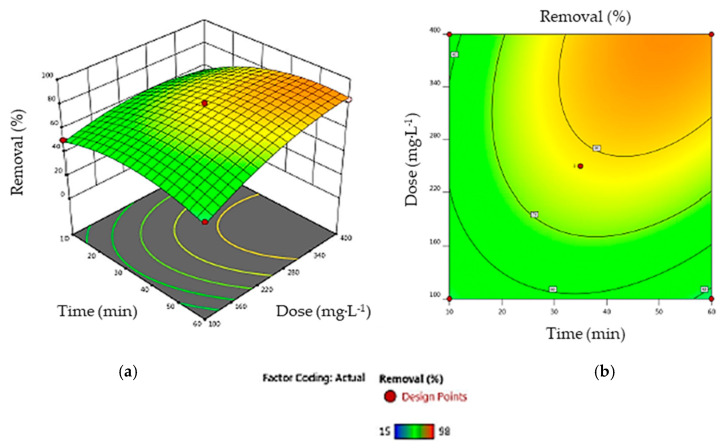
(**a**) The response surface and (**b**) the contour map showing the interaction of contact time and AC dosage (input parameters *X*_1_ and *X*_3_ in Equation (11), respectively) on dye removal.

**Figure 13:**
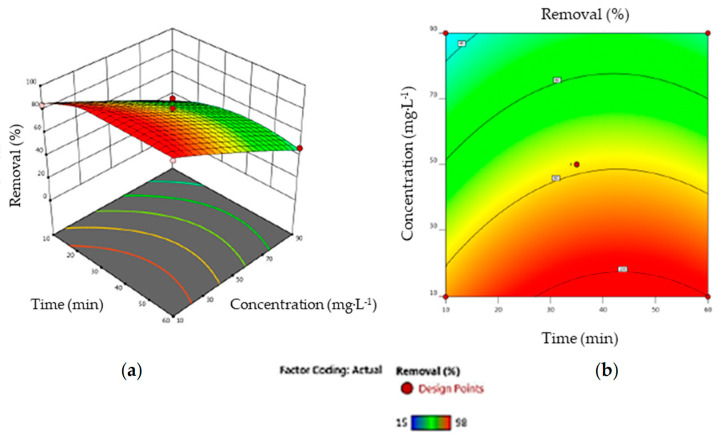
(**a**) The response surface and (**b**) the contour map showing the interaction of the AO7 concentration and contact time (input parameters *X*_2_ and *X*_1_ in Equation (11), respectively).

1. **Dye concentration and adsorbent dosage**. Figure 11 illustrates the interaction effects of the dye concentration and adsorbent dose on the removal efficiency at a fixed time of 35 min. This shows that better responses were observed with higher adsorbent masses. This result shows the importance of the factors involving the input parameter *X_3_* in Equation (11). Increasing the amount of AC enhances the availability of active adsorption sites for dye binding, thus promoting the removal process [42]. On the other hand, increasing the dye concentration from 10 to 90 mg·L^−1^ while maintaining the AC dose at 0.250 g·L^−1^ results in a reduction in removal from 98% to 60%. The high removal efficiency of adsorbates at low concentrations can be attributed to the large number of adsorption sites available. This allows greater accessibility and facilitated adsorption, leading to the removal of a greater proportion of adsorbate from solution. It is important to note that the number of available sites is a major factor in determining the adsorption efficiency *Y*, showing the antagonistic impact of input parameters *X*_2_ and *X*_3_ in Equation (11). As the initial concentration of AO7 (*X*_2_) increases, the number of available adsorption sites decreases, reducing the efficiency of adsorption removal [57].
2. **Adsorbent dose and contact time.** The 3D response surface and contour map, illustrating the interaction effects of the contact time (*X*_1_) and adsorbent dose (*X*_3_) on dye removal efficiency (*Y*) at a fixed dye concentration (50 mg·L^−1^), are shown in Figure 12. It can be seen that, for an AC adsorbent dose of less than 0.250 g·L^−1^, the contact time has a negligible effect on AO7 retention, as the available active sites are few in number and rapidly saturated. On the other hand, at a dose higher than 0.250 g·L^−1^, AO7 retention increases with the contact time up to 40 min, when retention becomes stable as all active sites are saturated and equilibrium is reached [58].
3. **Contact time and dye concentration**. The 3D graph and contour map shown in Figure 13 illustrate the combined influence of dye concentration (*X*_2_) and contact time (*X*_1_) on the removal efficiency (*Y*). The experiment was carried out with a constant AC dosage of 0.250 mg·L^−1^. The contour map reveals an almost constant removal efficiency, indicating that AO7 adsorption remained relatively unchanged with the varying contact times over the range studied. This lack of significant change in removal with the contact time suggests rapid adsorption kinetics. However, increasing the dye concentration and maintaining a constant contact time leads to local saturation of the most easily accessible active sites. This phenomenon causes the driving force to remain at the same level until the adsorption process reaches equilibrium [59]. In general, the interaction of the dye concentration and time of contact has a negative effect on the response variable.

#### 3.4.3. Process Optimization

The optimum conditions in the range studied predicted by the model to ensure maximum AO7 removal were an adsorbent dose of 0.285 g·L^−1^, a dye concentration of 20 mg·L^−1^ and a contact time of 40 min. These conditions were expected to lead to a dye removal efficiency of 98%. An experiment was carried out using these specified parameters. The obtained results showed a discoloration rate of 97.6%, corresponding closely to the value predicted by the model. The agreement between experimental and predicted values confirms the accuracy and reliability of the customized model. Consequently, the model is considered a reliable tool for predicting and monitoring AO7 removal efficiency using AC.

**Figure 14:**
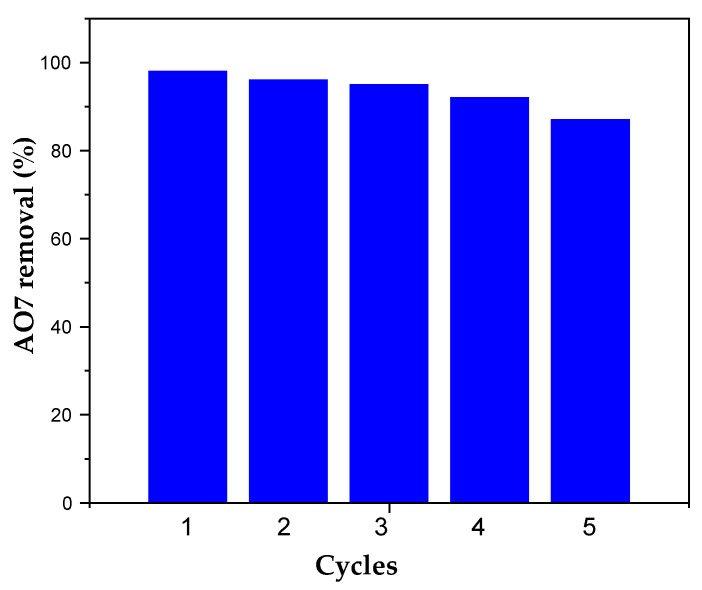
Removal rate of AO7 and recovery efficiency of AC after 5 adsorption/regeneration cycles.

To test the reusability of AC after dye removal, desorption experiments were carried out with 50 wt.% ethanol at room temperature for 2 h. The adsorbent obtained was then recycled four times to remove AO7 (Figure 14).

As can be seen, the results show that AC after regeneration can be reused with a capacity loss of only a few percent for the first three cycles. A more significant drop was observed for the subsequent cycles. This could be due to irreversible adsorption on high-affinity sites. Dye accumulation on the adsorbent surface would partially limit the availability of other adsorption sites. However, given the low cost of this adsorbent, reuse over a small number of cycles should be sufficient for large-scale use.

### 3.5. Comparative Study

Table 12 compares the efficiency of the different adsorbents used to remove AO7 dye from aqueous solution reported in the literature [18,43,58,60,61,62,63]. As shown in this survey, the AC obtained from the SCGs had an excellent affinity for AO7 dye removal (in terms of adsorption capacity and kinetics) and compared favorably with the other low-cost activated carbon and bio-adsorbents. The dose of adsorbent required is comparatively low (0.285 g·L^−1^), and the contact time is equally short (40 min), which may be explained by the high accessibility of adsorption sites despite the low specific surface area (BET) of AC compared to activated carbon produced at higher temperatures. These operating conditions are very attractive and advantageous for a dye removal process using adsorption on activated carbon. Consequently, AC prepared from SCGs according to the procedure described in this work is a promising adsorbent with great potential for the decolorization of wastewater from the textile industry.

## 4. Conclusions

A simple and reproducible procedure for preparing activated carbon (AC) from used coffee grounds was successfully developed involving chemical activation with a small amount of H_3_PO_4_ at the relatively low temperature of 600 °C, making it an inexpensive material. It was found that the two batches of AC prepared exhibited excellent and similar adsorption properties for AO7 removal from aqueous solutions. A comprehensive adsorption study was conducted, including kinetics, thermodynamics, mechanism and process optimization.

Analysis of surface chemical functions by FTIR and a thermodynamic study revealed that AO7 adsorbs to AC by spontaneous physisorption involving mainly hydrogen bonds and π–π interactions. The modeling of AO7 adsorption by AC showed that experimental kinetic and isotherm data fitted best with the pseudo-second-order model and the Langmuir model, respectively. To optimize AO7 removal by AC, experimental design methodology was applied, allowing the influence of specific parameters such as contact time, dye concentration and adsorbent quantity to be determined. The optimal design model fitted with the experimental data showed a high coefficient of determination (*R*^2^ = 0.9918) and an adjusted *R*^2^ coefficient of 0.9770, indicating that it is a robust predictive model. In addition, the statistical significance was validated by a *p*-value associated with the model of less than 0.05. The optimal conditions for the nearly complete removal of AO7 (98%) at a concentration of 20 mg·L^−1^, predicted by the model and experimentally tested, required a contact time of 40 min and an adsorbent dosage of 0.285 g·L^−1^.

The adsorption of dyes contained in textile industry wastewater using inexpensive, locally available activated carbon prepared from spent coffee grounds therefore appears to be a promising and effective treatment for pollution challenges, with a strong potential for scale-up. A comprehensive study of the adsorption properties with real effluents is currently underway and will be published in the near future.

## Figures and Tables

**Table 1:** Main characteristics of AO7.

Chemical Structure	Molecular Formula	Molecular Weight (g·mol^−1^)	Solubility in Water (g·L^−1^)	Melting Point (°C)	λ Max (nm)
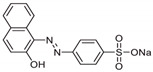	C_16_H_11_N_2_SO_4_Na	350.32	116	164	483

**Table 2:** Kinetic models.

Kinetic Model	Equation	
Pseudo-first-order	$\log(q_{e}-q_{t})=\log q_{e}-\frac{K_{f}\cdot t}{2303}$	(3)
Pseudo-second-order	$\frac{t}{q_{t}}=\frac{1}{K_{s}\cdot q_{e}^{2}}+\frac{t}{q_{e}}$	(4)

where *q_t_* and *q_e_* (mg·g^−1^) are the adsorption capacity at time *t* and equilibrium, respectively, and *K_f_* and *K_s_* are the pseudo-first-order (min^−1^) and pseudo-second-order (g (mg·min)^−1^) adsorption rate constants, respectively.

**Table 3:** Isotherm models.

Isotherm Model	Equation	
Langmuir	$\frac{C_{e}}{q_{e}}=\frac{1}{K_{L}{\cdot q}_{max}}+\frac{C_{e}}{q_{max}}$	(5)
Freundlich	$\log q_{e}=\log k_{f}+\frac{1}{n}\cdot \log C_{e}$	(6)

where *q_max_* and *q_e_* (mg·g^−1^) are the maximum monolayer adsorption capacity of the adsorbent and at equilibrium in Equations (5) and (6), respectively, *C_e_* (mg·L^−1^) the equilibrium value of the initial concentration, *K_L_* the Langmuir constant (mg·L^−1^) and *K_F_* the Freundlich constant.

**Table 4:** Thermodynamic parameters.

Thermodynamic Parameters	Equation	
Gibbs free energy	*K*_*c*_ = *K*_*L*_·1000·55.51·*M*w	(7)
	*ΔG*° = −*RT* Ln *K_c_*	(8)
Enthalpy Entropy	$\mathrm{Ln}K_{c}=(\frac{\Delta S{}^{\circ}}{R})-(\frac{\Delta H{}^{\circ}}{R})\cdot \frac{1}{T}$	(9)

where *K_c_* (dimensionless) is the equilibrium constant; *K_L_* (L·mg^−1^) the Langmuir constant; 55.51 the number of moles of pure water per liter (mol·L^−1^); *M*w (g·mol^−1^) the molecular weight of the dye; *T* (°K) is the absolute temperature; *R* the ideal gas constant (8.314 J·mol^−1^·K^−1^); and *ΔG°* (kJ·mol^−1^) is the Gibbs free energy change, *ΔH°* (kJ·mol^−1^) the enthalpy change and *ΔS°* (J·mol^−1^·K^−1^) the entropy change.

**Table 5:** BET measurements of AC.

	*S_BET_* (m^2^·g)	*V_total_* (cm^3^·g)	*D_m_* (nm)
AC (from SCG)	29.38	0.067	5.5

**Table 6:** Kinetic parameters for AO7 adsorption.

Pseudo-First-Order			Pseudo-Second-Order		
*K_f_* (min^−1^)	*q_e_* (mg·g^−1^)	R^2^	*K_s_* (mg·(g·min)^−1^)	*q_e_* (mg·g^−1^)	R^2^
0.12	46.99	0.898	0.003	55.5	0.999

**Table 7:** Isotherm modeling parameters for AO7 adsorption.

	Langmuir				Freundlich		
T (K)	*K_L_* (L·mol^−1^)	*q_max_* (mg·g^−1^)	*R* ^2^	*R_L_*	*K_F_* (L·g^−1^)(L·mg^−1^)^1/n^	1/n	*R* ^2^
298	0.154	151.51	0.9905	0.178	64.24	0.192	0.9852
321	0.203	153.85	0.9817	0.247	65.39	0.203	0.9369
339	0.349	156.25	0.9904	0.223	73.48	0.204	0.9579

**Table 8:** Thermodynamic parameters for AO7 adsorption on AC.

Thermodynamic Parameters	*ΔG°* (kJ·mol^−1^)			*ΔH°* (kJ·mol^−1^)	*ΔS°* (J·(mol·K)^−1^)
T (°K)	298	321	339		
	−7.43	−9.17	−10.53	15.12	75.65

**Table 9:** Box–Behnken design and comparison of experimental- and model-predicted results for AO7 adsorption using AC.

Test Number	Factors			Response *Y*		Residual (% Removal)
	*X*_1_ (min)	*X*_2_ (mg·L^−1^)	*X*_3_ (g·L^−1^)	Experimental (% Removal)	Predicted (% Removal)	
1	60 (+1)	50 (+0)	0.100 (−1)	49.3	47.15	2.15
2	35 (+0)	50 (+0)	0.250 (+0)	84.0	85.45	−1.45
3	60 (+1)	10 (−1)	0.250 (+0)	97.2	97.19	0.0063
4	10 (−1)	90 (+1)	0.250 (+0)	47.0	45.55	1.45
5	35 (+0)	50 (+0)	0.250 (+0)	84.0	85.44	−1.44
6	10 (−1)	50 (+0)	0.100 (−1)	36.3	34.09	2.21
7	60 (+1)	90 (+1)	0.250 (+0)	51.0	49.56	1.44
8	35 (+0)	90 (+1)	0.400 (+1)	54.7	56.87	−2.17
9	10 (−1)	10 (−1)	0.250 (+0)	77.7	78.02	−0.3233
10	60 (+1)	50 (+0)	0.400 (+1)	98.0	94.39	3.61
11	35 (+0)	10 (−1)	0.100 (−1)	67.0	67.01	−0.0063
12	35 (+0)	10 (−1)	0.400 (+1)	98.0	100.16	−2.16
13	35 (+0)	50 (+0)	0.250 (+0)	81.1	78.02	3.1
14	35 (+0)	90 (+1)	0.100 (−1)	15.0	18.61	−3.61
15	10 (−1)	50 (+0)	0.400 (+1)	75.3	78.02	−2.72

**Table 10:** Analysis of variance for the adsorption of AO7 dye by AC.

Source	Sum of Squares	Degree of Freedom	Mean of Squares	*F*-Value	*p*-Value		*R* ^2^	*R* ^2^ *_adj_*
Model	8485.68	9	942.85	66.94	0.0001	significant	0.9918	0.9770
Error	17.39	2	8.69					

**Table 11:** Estimated polynomial coefficients for the adsorption of AO7 dye by AC.

Coefficient	Estimated Coefficient	*F*-Value	*p*-Value
a_0_	78.02		
a_1_	6.54	24.32	0.0044
a_2_	−26.49	398.7	<0.0001
a_3_	11.40	73.82	0.0004
a_1_a_2_	−0.813	0.188	0.6831
a_1_a_3_	7.75	17.04	0.0091
a_2_a_3_	12.80	46.53	0.001
a_1_^2^	−10.63	29.61	0.0028
a_2_^2^	−1.08	0.307	0.6031
a_3_^2^	−7.64	15.3	0.0113

**Table 12:** Comparative study of the adsorption of AO7 dye on various bio-sorbents from the literature.

Bio-Sorbent	Experimental Conditions				Results		Lit.
	pH	AO7 (mg·L^−1^)	Adsorbent Dose (g·L^−1^)	Contact Time (min)	Removal (%)	Capacity (mg.g^−1^)	
Bifurcaria bifurcate activated carbon	7.5	10	0.2	120	88.8	82.56	[18]
Activated carbon based on grape marc	2	150	2	180	-	140.5	[43]
Aloe vera leaves	2	50	2.5	360	-	15.9	[58]
Coconut coir activated carbon	3	40	6	120	99.5	13.16	[60]
Activated carbon from Pisum sativum pods	1.5	100	2	95.7	~100	473.93	[61]
Spent brewery grains	4.5	60	3.75	60	>90	30.5	[62]
Activated carbon from Casuarina wood	2.8	25	2	90	83.4	9.51	[63]
AC from SCG	7.5	20	0.285	40	98	119.51	^1^

^1^ This study.

## Data Availability

Dataset available on request from the corresponding authors.
